# The association between expressions of Ras and CD68 in the angiogenesis of breast cancers

**DOI:** 10.1186/s12935-015-0169-1

**Published:** 2015-02-07

**Authors:** Wei Li, Rong-Rui Liang, Chong Zhou, Meng-Yao Wu, Lian Lian, Gao-Feng Yuan, Ming-Yun Wang, Xin Xie, Liu-Mei Shou, Fei-Ran Gong, Kai Chen, Wei-Ming Duan, Min Tao

**Affiliations:** Department of Oncology, the First Affiliated Hospital of Soochow University, Suzhou, 215006 Jiangsu Province People’s Republic of China; Department of Radiation Oncology, the Central Hospital of Xuzhou, Xuzhou, 221009 Jiangsu Province People’s Republic of China; Department of Oncology, Suzhou Xiangcheng People’s Hospital, Suzhou, 215131 Jiangsu Province People’s Republic of China; Department of Oncology, Sihong People’s Hospital, Sihong, 223900 Jiangsu Province People’s Republic of China; Department of Oncology, Nanjing Gaochun People’s Hospital, Gaochun, 211300 Jiangsu Province People’s Republic of China; Department of Oncology, Affiliated Hospital of Xuzhou Medical College, Xuzhou, 221006 Jiangsu Province People’s Republic of China; Department of Oncology, the first Affiliated Hospital of Zhejiang Chinese Medicine University, Hangzhou, 310006 Zhejiang Province People’s Republic of China; Department of Hematology, the First Affiliated Hospital of Soochow University, Suzhou, 215006 Jiangsu Province People’s Republic of China; Jiangsu Institute of Clinical Immunology, Suzhou, 215006 Jiangsu Province People’s Republic of China

**Keywords:** Breast cancer, Ras, TAM, CD34, Angiogenesis

## Abstract

**Objective:**

Angiogenesis is a critical step of breast cancer metastasis. Oncogenic Ras promotes the remodeling of cancer microenviroment. Tumor-associated macrophages (TAMs) are a prominent inflammatory cell population emerging in the microenviroment and facilitating the angiogenesis and metastasis. In the present study, we tried to investigate the relationship between the expression of Ras and infiltration of TAM, both of which could further promote angiogenesis.

**Methods:**

Expressions of Ras, CD68 and CD34 were assessed by immunohistochemistry. The infiltration of macrophages was evaluated by counting the number of CD68^+^ cells. Vessel endothelial cells were defined as CD34^+^ cells. Angiogenesis vascularity was defined by microvessel density (MVD) assay through counting the number of vessels per field counted in the area of highest vascular density. The Kaplan–Meier survival analysis was used to estimate the overall survival (OS). Macrophages were derived from monocytes in the presence of macrophage colony-stimulating-factor (MCSF). Breast cancer cells were treated with macrophage-conditioned medium (MCM) and tested the expressions of K-, H- and N-Ras by using realtime-PCR.

**Results:**

Ras positive status was correlated with ER, PR and Her-2 positivity, larger tumour size and lymph node metastasis, as well as higher TNM stages. A higher number of CD68^+^ cells was correlated with larger tumour size, higher TNM stages and Her-2 positivity. Both Ras positivity and infiltration of CD68^+^ macrophages correlated with poor OS. The number of CD68^+^ cells was positively correlated with the expression of Ras. Treatment with MCM did not up-regulate but repressed the expression of Ras. Both up-regulation of Ras and infiltration of TAMs correlated with increased MVD.

**Conclusion:**

Expression of Ras and infiltration of TAM were positively correlated, and both participated in angiogenesis. Elevated Ras could be responsible for the infiltration of TAM.

## Introduction

Breast cancer is one of the most frequent causes of cancer mortality in females in developed countries. Although early detection, precise resection using wide margins, and systematic adjuvant therapy have improved survival, recurrence and metastasis remains the leading cause of breast cancer-related mortality [1-4].

Angiogenesis, the process of forming new blood vessels from existing vascular networks, is a critical event which is essential for the growth and persistence of solid tumors and their metastasis [5]. Data from experimental and clinical studies indicate that breast cancer is an angiogenesis-dependent tumor. It has been suggested that the intensity of angiogenesis may be inversely correlated with the time of survival of patients with invasive breast cancer [6]. Several methods, such as visual vascular grading, manual counting of microvessels in defined microscopic field areas termed as microvessel density (MVD), and the Chalkley counting method, have been applied to quantify tumour vascularity by using immunostaining for different types of endothelial markers, such as CD31 and CD34 [7,8]. Many literatures have concluded that the MVD assay, developed by Weidner et al. [9,10], is a reliable method for investigating the prognostic value of angiogenesis in patients with breast cancer [8].

It has been increasingly recognized that tumor microenvironment plays an important role in angiogenesis [11]. Tumor-associated macrophages (TAMs) are a prominent inflammatory cell population in many tumor types residing in both perivascular and avascular, hypoxic regions of these tissues. Analysis of TAMs in human tumor biopsies has shown that they express a variety of tumor-promoting factors and evidence from transgenic murine tumor models has provided unequivocal evidence for the importance of these cells in driving angiogenesis, lymphangiogenesis, immunosuppression, and metastasis [12]. It has been well accepted that TAMs are generally recruited from blood monocytes by diverse chemokines such as CCL2 (MCP-1), CCL5, CCL7, interleukin 8 (IL-8, CXCL8) and CXCL12, followed by migration to diverse areas of the tumor microenvironment and differentiation according to surrounding cellular or environmental stimuli [13].

Ras protein family members, composed of K-, H- and N-Ras, belong to a class of protein called small GTPase, and are involved in cellular signal transduction. As these signals result in cell growth and division, overactive Ras signaling can ultimately lead to carcinogenesis [14]. Previous study proved that activation of oncogenic mutant K-Ras could result in up-regulation of CCL2 (MCP-1) [15]. Activation of Ras pathway also increased expression of CCL5 [16,17]. Moreover, oncogenic Ras upregulates the expression of IL-8 [14], while shRNA-mediated K-Ras knockdown induced significant down-regulation of IL-8 [18]. These investigations suggested that the recruitment of TAM could be a consequence of activation of Ras pathway. However, the relationship between Ras expression and TAM infiltration has not been investigated in breast cancers. In the present study, we investigated the association between TAM marker, CD68, and Ras expression in breast cancer specimens. The participation of TAM and Ras in angiogenesis was also investigated.

## Methods

### Patients and tissue material

The material consisted of 120 female breast cancer patients whose tissue samples were available (mean age at surgery was 51.4 years, range 30–82 years) and who were operated and treated during 2007.01-2009.9. All human tissue samples were obtained and handled in accordance with an approved Institutional Review Board application (the Committee on Medical Ethics, the First Affiliated Hospital of Soochow University). Tumour characteristics were obtained from the pathology database (Table 1). None of the patients received radiation or chemotherapy before operation. All breast tumours were invasive carcinomas. All of the tumors were classified according to the International Union Against Cancer (UICC) tumor/lymph node/metastasis (TNM) classification system [19]. Patients received systemic adjuvant therapy after surgery according to NCCN (National Comprehensive Cancer Network) breast cancer clinical practice guidelines [20]. Generally, patients with low risk (Node-negative, ER/PR positive, T ≤ 1 cm, grade 1, no lymphovascular invasion, Her-2/neu negative, age ≥ 35) received no adjuvant therapy or received endocrine therapy only; patients with intermediate risk (Node-negative and at least one of the following: T > 2 cm, grade > 1, lymphovascular invasion, age < 35, Her-2/neu positive; or one to three nodes positive and Her/2-neu negative) received adjuvant chemotherapy, adjuvant chemotherapy followed by endocrine therapy, or endocrine therapy only; patients with high risk (one to three nodes positive and Her/2-neu positive; or ≥ four nodes positive) received adjuvant chemotherapy, or adjuvant chemotherapy followed by endocrine therapy. The adjuvant chemotherapy regimens include FAC/CAF (Fluorouracil/doxorubicin/cyclophosphamide), FEC/CEF (Fluorouracil/epirubicin/cyclophosphamide), AC (Doxorubicin/cyclophosphamide), CMF (Cyclophosphamide/methotrexate/fluorouracil), TAC (Docetaxel/doxorubicin/cyclophosphamide), TC (Docetaxel/cyclophosphamide), AC → T (Doxorubicin/cyclophosphamide, followed by paclitaxel), and AC → T + H (Doxorubicin/cyclophosphamide, followed by paclitaxel plus trastuzumab). Patients who suffered from tumor recurrence received palliative chemotherapy by using single agents or combination regimens. Single agents included doxorubicin, paclitaxel, capecitabine, gemcitabine, vinorelbine, and cisplatin. Combination regimens included FAC/CAF (Fluorouracil/doxorubicin/cyclophosphamide), FEC/CEF (Fluorouracil/epirubicin/cyclophosphamide), AC (Doxorubicin/cyclophosphamide), EC (epirubicin/cyclophosphamide), CMF (Cyclophosphamide/methotrexate/fluorouracil), GT (gemcitabine/paclitaxel). Application of trastuzumab depended on Her-2 status. Patients were followed regularly for 5 years. The prognostic analyses were performed regarding overall survival (OS).

**Table 1:** **Clinicopathologic characteristics of tumour**

**Variable**	**Number of patients (%)**
Number of the patients	120
Length	
<2 cm	12 (10%)
≥2 cm	108 (90%)
lymph node status	
N0	48 (40%)
≥N1	72 (60%)
TNM staging	
I	8 (6.67%)
II	73 (60.83%)
III	39 (32.5%)
Estrogen receptor status (ER)	
Negative	60 (50%)
Positive	60 (50%)
Progesterone receptor status (PR)	
Negative	87 (72.5%)
Positive	33 (27.5%)
Her-2	
IHC negative (0 and 1+)	68 (56.67%)
IHC positive (2+ and 3+)	52 (43.33%)

### Immunohistochemistry

All resection specimens in this study were fixed in 10% buffered formalin and paraffin embedded by routinely processing. Sections were cut at a thickness of 4 μm, heated at 60°C for 30 min, then deparaffinized and hydrated through a series of xylene and alcohol baths before staining. The slides were microwaved with antigen retrieval solution (citrate buffer, pH 6.0, containing 0.3% trisodium citrate and 0.04% citric acid) for 5 min. After replenishment of this solution, the slides were microwaved again for an additional 5 min and then allowed to cool for 20 min. The sections were then rinsed in PBS (phosphate-buffered saline), and immersed in 3% H_2_O_2_ for 15 minutes to block the endogenous peroxidase. Thereafter, the sections were incubated with 10% BSA (bull serum albumin) at room temperature for 60 minutes to block nonspecific antibodies. Immunohistochemical staining was performed with rabbit anti-Ras antibody (Abcam, ab108602), mouse anti-CD68 antibody (Abcam, ab49777), or rabbit anti-CD34 antibody (Abcam, ab81289) respectively at room temperature for 2 h. After incubation with the corresponding secondary antibodies for 20 min, the bound complex was visualized by using the SuperPicTure polymer detection kit (No.87-8963; Invitrogen).

### Evaluation of immunostaining

All the staining results were evaluated by two independent researchers. All analyses were performed blind with respect to the clinical outcomes. A positive stain for Ras was defined as brown stain seen in the cytoplasm.

The infiltration of macrophages was evaluated by counting the number of CD68^+^ cells as previously described [21]. Briefly, the five most representative high-power fields (×400 magnification) per slide were selected. Tumour-infiltrating macrophages were large irregular cells with oval to round nuclei and fine processes that showed strong cytoplasmic staining (sometimes granular) but no nuclear staining for CD68. The number of CD68^+^ nucleated cells was counted manually and expressed as cells per mm^2^. To evaluate the validity of the analysis, the area measurement and counting were repeated 4 weeks later.

Angiogenesis vascularity was defined as the number of vessels per field counted in the area of highest vascular density, termed as microvessel density (MVD) [9,10]. Endothelial cells were marked with anti-CD34 antibody. CD34 antigen was localized in the cytoplasm and cellular membrane of vascular endothelial cells. Single endothelial cells, endothelial cell clusters, and microvessels in the tumors, clearly separated from adjacent microvessels, were counted. Peritumoral vascularity and vascularity in areas of necrosis were not scored. A vascular lumen was not a requirement for a structure to be counted as a microvessel. Branching structures were counted as one, unless there was a break in the continuity of the vessel, in which case it was counted as two distinct vessels. Areas with a higher density of CD34^+^ cells and cell clusters relative to adjacent areas were classified as ‘hot spots’. The slides were initially screened at low power to identify the areas with the highest number of microvessels or vascularity hot spots. Microvessels were counted in 400× magnification fields. MVD was defined as the number of manually counted vessel profiles per mm^2^ taken as the average from the three hot-spot counts.

### Cell culture

MCF-7 and MDA-MB-231 breast cancer cells were obtained from American Type Culture Collection (Manassas, VA, USA) and maintained in RPMI-1640 (Gibco, Grand Island, NY, USA) medium supplemented with 10% fetal calf serum (FCS; Hyclone, Logan, UT, USA), 100 U/ml penicillin and 100 mg/ml streptomycin at 37°C in a humidified atmosphere with 5% CO_2_. The cells were passaged every 2–3 days to maintain exponential growth.

### Macrophage preparation and culture

Buffy coats that contain mononuclear cells were collected from the blood of healthy individual donors at the First Affiliated Hospital of Soochow University with an approved Institutional Review Board application (the Committee on Medical Ethics, the First Affiliated Hospital of Soochow University). Primary blood monocytes were isolated by density-gradient centrifugation through Ficol/Hypaque (Amersham Bioscience, Piscataway, NJ), suspended (8 × 10^6^ cells/ml) in DMEM medium (Gibco) with 10% heat-inactivated human serum (Sigma, St. Louis, MO), and seeded in culture dishes. After incubation for 2 h at 37°C, adherent cells were cultured in medium supplemented with 40 ng/ml human MCSF (PeproTech Inc, Rocky Hill, NJ). Cells were allowed to differentiate for 7 days in the presence of MCSF. On day 7, fresh medium without MCSF was added to the cells, and the cells continued to culture for 24 h. The culture medium was collected, centrifuged, stored in aliquots at −80°C, and defined as macrophage-conditioned medium (MCM).

### Real-time PCR

Total RNA was extracted using Trizol reagent (Invitrogen, Valencia, CA, USA) according to the manufacturer’s protocol. After spectrophotometric quantification, 1 μg of total RNA in a final volume of 20 μl was used for reverse transcription with AMV reverse transcriptase (Promega, WI, USA) according to the manufacturer’s protocol. Aliquots of cDNA, which corresponded to equal amounts of RNA, were used for the quantification of mRNA by real-time PCR using a TL988 Real-time Quantitative PCR Detection System (TianLong Science and Technology, Xi’An, Shanxi, China). The reaction system (20 μl) contained the corresponding cDNA, forward and reverse primers, and SYBR Green PCR master mix (Roche, Indy, IN, USA). All the data were analyzed using β-actin gene expression as an internal standard. The specific primers were as follows: (1) K-Ras: forward, 5′-AGAGTGCCTTGACGATACAGCT-3′, reverse, 5′-CAGTCCTCATGTACTGGTCCCTC-3′, product, 177 bp; (2) H-Ras: forward, 5′-CACCAGTACAGGGAGCAGATCA −3′, reverse, 5′-TGAGCCTGCCGAGATTCCA-3′, product, 113 bp; (3) N-Ras: forward, 5′-TACATGAGGACAGGCGAAGG-3′, reverse, 5′-TGGCAATCCCATACAACCCT-3′, product, 345 bp; and (4) β-actin: forward, 5′-TCATGAAGTGTGACGTGGACAT-3′, reverse, 5′-CTCAGGAGGAGCAATGATCTTG-3′, product, 158 bp.

### Statistical analysis

Each experiment was performed at least in triplicate. Statistical analysis was performed using SAS 9.2 software (SAS Institute Inc., Cary, NC, USA). The correlations between Ras, CD68^+^ cell infiltration and clinicopathologic features were analyzed using Spearman’s rank test. The association between CD68^+^ cell number and MVD numbers were also performed using Spearman’s rank test. Chi-square tests were used for comparison of Ras expression in various stages. For analysis of survival data, Kaplan–Meier curves were constructed, and statistical analysis was carried out using the log-rank test. OS was defined as the time from beginning of surgery to death from any cause or the last date of follow-up. For the Ras gene expression data, statistical analyses were carried out with unpaired Student’s *t*-test. All values of *P* < 0.05 were considered statistically significant, and all tests were two-sided.

## Results

### Ras expressions is associated with clinical outcomes in breast cancers

**Figure 1:**
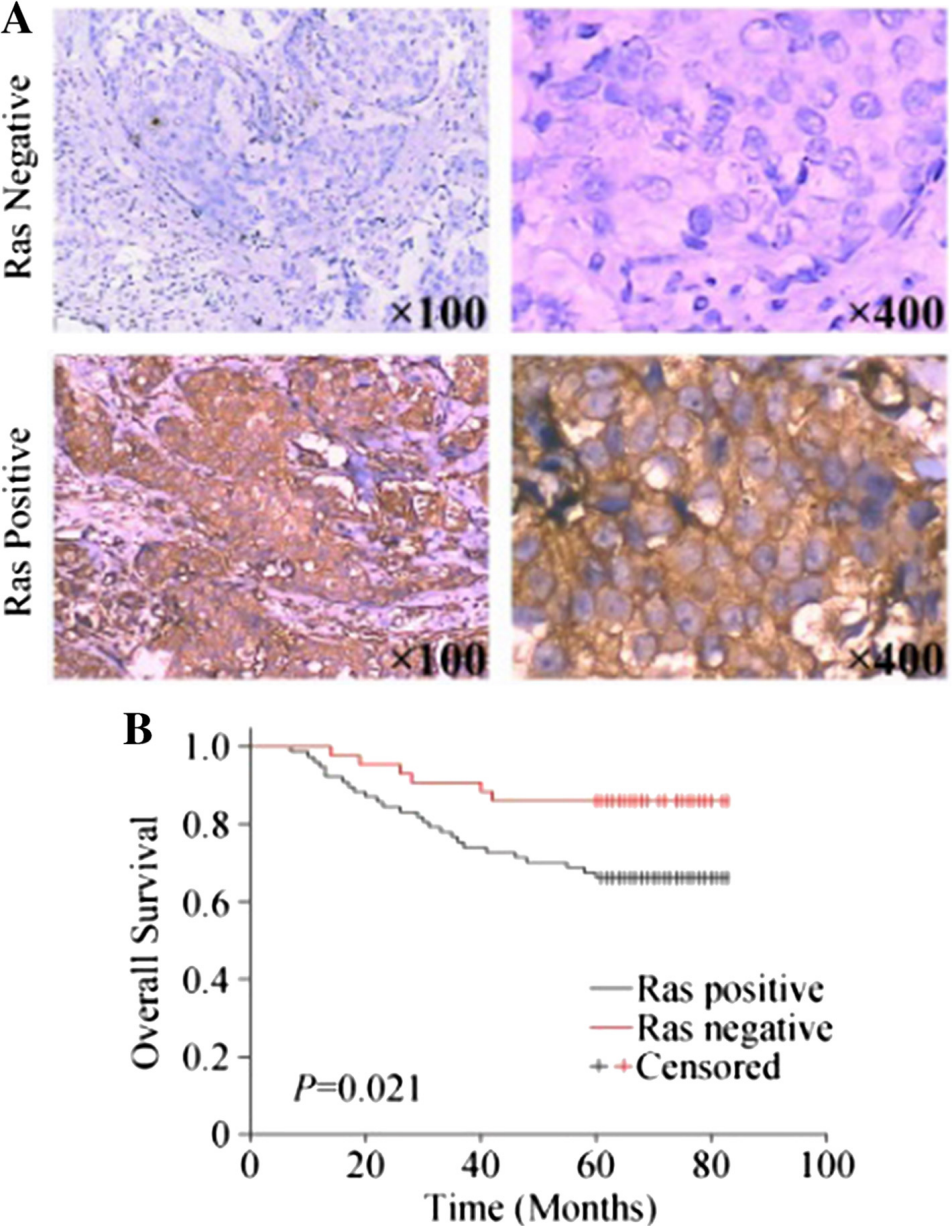
**Effects of of Ras expression on overall survival in breast cancers. (A)** Expression of Ras in breast cancers. **(B)** Kaplan—Meier plot of overall survival (OS) stratified by Ras expression.

Among the 120 samples, Ras could be detected in 77 specimens (64.17%). In particular, the Ras positive rate was 20.00% for patients with stage I disease, compared with 54.79% for patients with stage II and 89.74% for patients with stage III disease (*P* <0.0001, Table 2). Moreover, a Ras positive status was correlated with ER, PR and Her-2 positivity, larger tumour size and lymph node metastasis, as well as higher TNM stages. Other histological features had no statistically significant association with Ras expression (Table 3). As shown in Figure 1B, positivity of Ras correlated with poor OS, suggesting the Ras status was an independent prognostic factor for survival.

**Table 2:** **Ras expression in various stages**

**Stage**	**n**	**Ras positive**	**Ras negative**	**Positive rate (%)**
I	8	2	6	20.00
II	73	40	33	54.79
III	39	35	4	89.74

Chi-square value = 19.22, *P* <0.0001.

**Table 3:** **Correlations between clinicopathologic features and Ras, CD68 or MVD**

**Clinicopathologic features**	**Ras**		**CD68**		**MVD**	
	**Correlation coefficient**	***P*** **value**	**Correlation coefficient**	***P*** **value**	**Correlation coefficient**	***P*** **value**
Age	−0.00524	0.955	−0.0861	0.349	−0.172	0.0599
Long diameter	0.396	0.00000879	0.355	0.0000777	0.168	0.0667
Tumor volume	0.513	0.00000000259	0.468	0.0000000938	0.393	0.0000104
Lymph node metastasis	0.348	0.000109	0.170	0.0633	0.000	1.000
TNM	0.400	0.00000714	0.263	0.00382	0.186	0.0418
ER	0.191	0.0366	−0.167	0.0689	0.233	0.0104
PR	0.227	0.0129	−0.0933	0.310	0.392	0.0000110
Her-2	0.303	0.000812	0.303	0.000814	0.101	0.272

### Infiltration of CD68^+^ macrophages predict outcomes in breast cancers

Infiltration of macrophage was evaluated using CD68 staining. CD68^+^ cells could be detected in 116 specimens (96.67%), with an average number of 20.44 and a median number of 11.6. Correlations between macrophage density and clinicopathological features were then analyzed. A higher number of CD68^+^ cells was correlated with larger tumour size, higher TNM stages and Her-2 positivity. Other histological features had no statistically significant association with CD68^+^ cell infiltration (Table 3).

**Figure 2:**
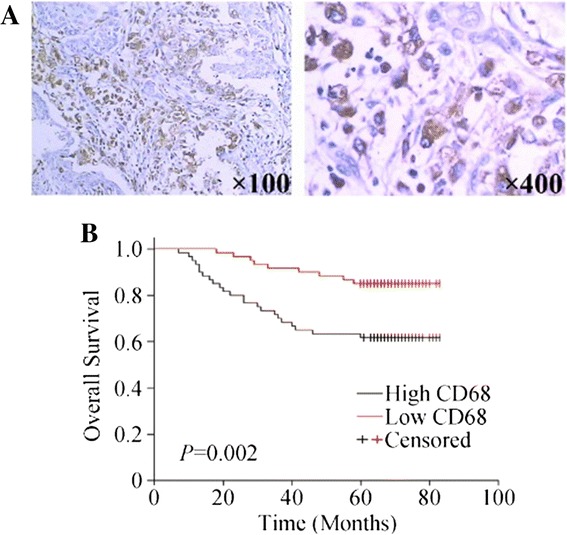
**Effects of of CD68** ^**+**^ **cell infiltration on overall survival in breast cancers. (A)** Infiltration of CD68^+^ cells in breast cancers. **(B)** Kaplan—Meier plot of overall survival (OS) stratified by CD68^+^ cell infiltration.

As shown in Figure 2B, infiltration of CD68^+^ macrophages correlated with poor OS, suggesting the enrichment of macrophage is predictive of poor prognosis and reduced survival in human breast cancer.

### Relationship between expressions of Ras and infiltration of CD68^+^ macrophages

The correlation between the expression of Ras and the number of CD68^+^ cells was than analyzed. The number of CD68^+^ cells in Ras negative group was 12.05 ± 18.44, compared with 25.13 ± 24.43 in Ras positive group. The number of CD68^+^ cells was positively correlated with the expression of Ras (r = 0.326, *P* = 0.000292).

**Figure 3:**
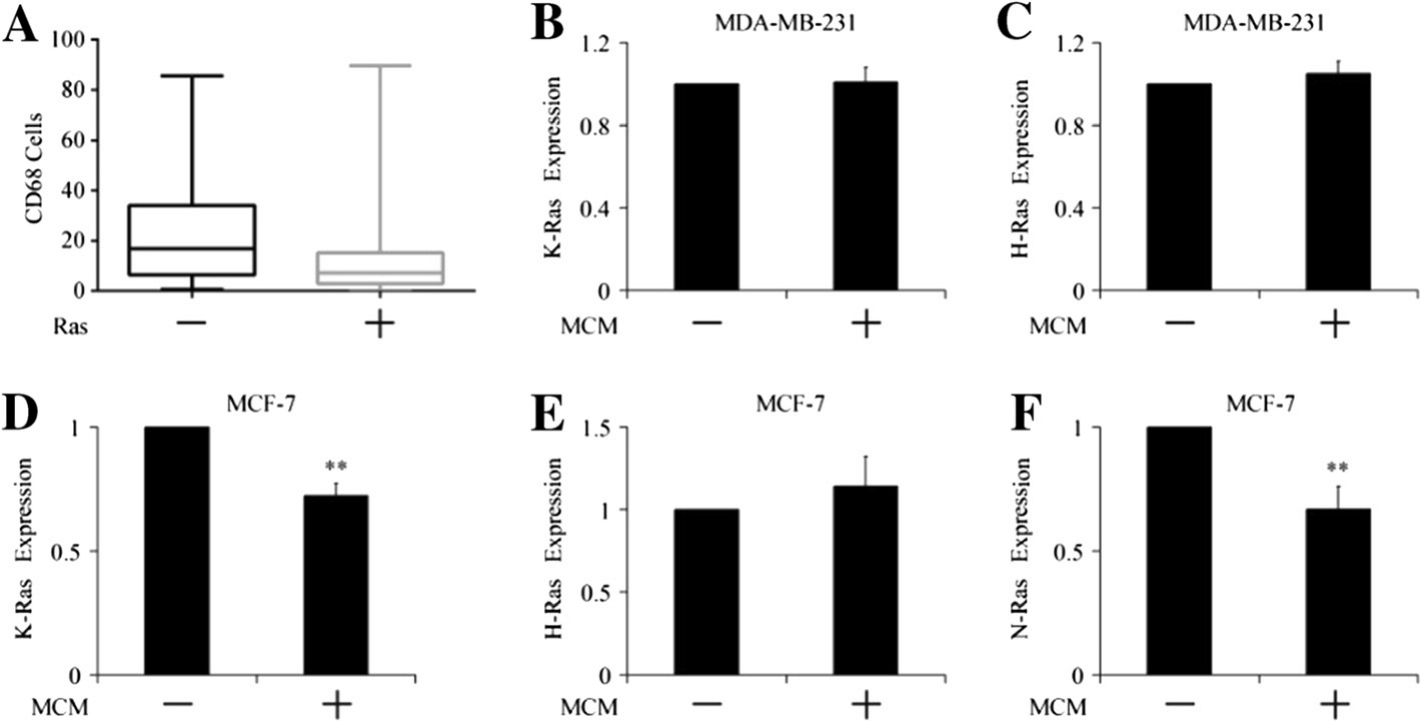
**Relationship between expressions of Ras and infiltration of CD68** ^**+**^ **cells. (A)** The association of Ras expression and CD68^+^ cells infiltration in breast cancer cells. **(B)**-**(F)** Expression of K-, H- and N-Ras in macrophage-conditional medium (MCM) treated MDA-MB-231 and MCF-7 cells. **P < 0.01 indicates significant differences from control group.

To find out the influence of macrophage on the expression of Ras, we treated breast cancer cells with macrophage-conditional medium (MCM), and evaluated the expression of K-Ras, N-Ras and H-Ras using Realtime-PCR. Expression of N-Ras was not found in MDA-MB-231 cells. As shown in Figure 3, treatment with MCM had no effects on the expressions of K-Ras or H-Ras in MDA-MB-231 cells. MCM had no effects on the expression H-Ras in MCF-7 cells neither, but repressed the expressions of K-Ras and N-Ras in MCF-7 cells. Thus, the infiltration of macrophage might be a negative regulator on the expression of Ras.

### Overexpression of Ras and infiltration of CD68^+^ macrophages correlated with angiogenesis

**Figure 4:**
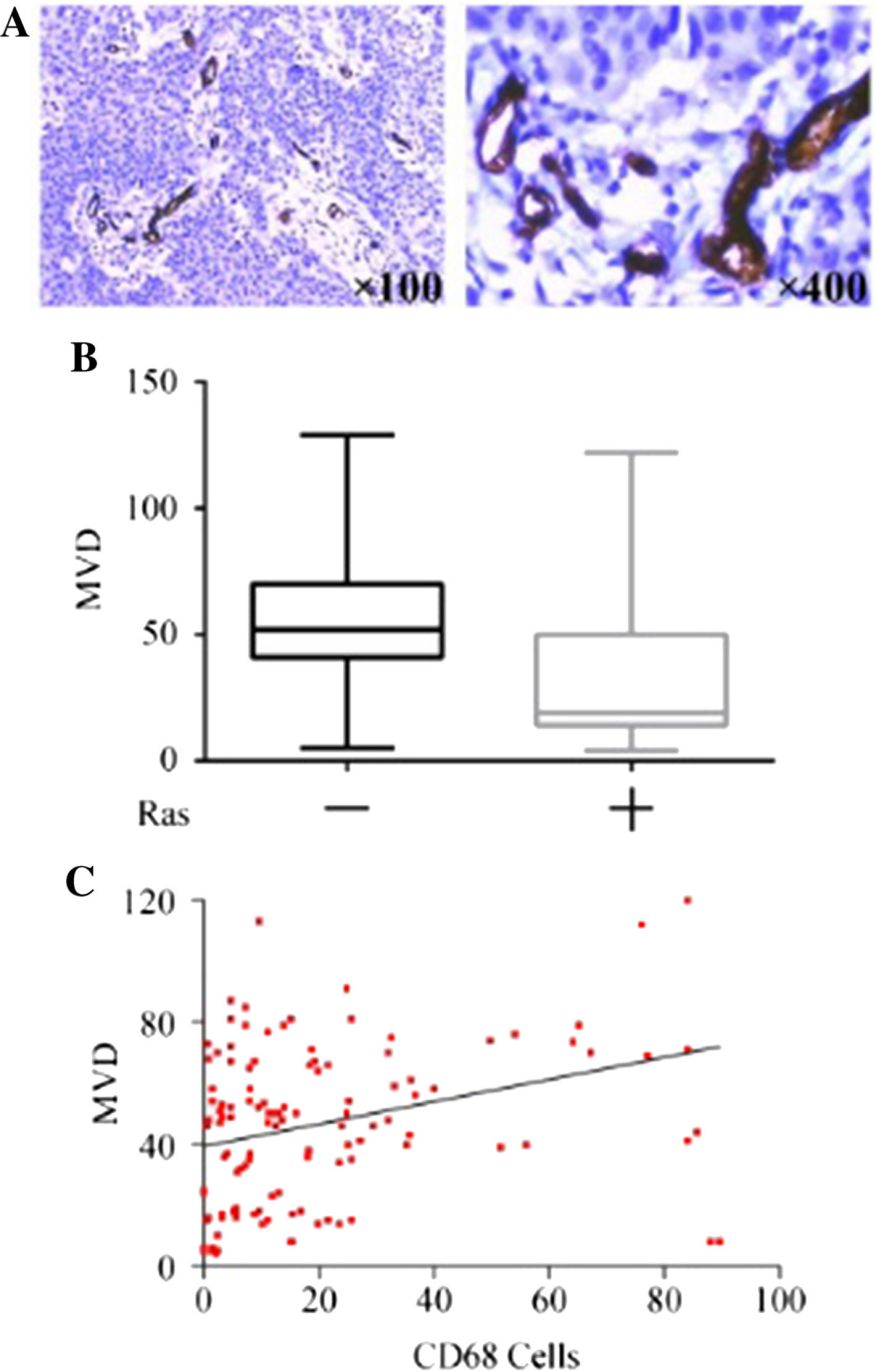
**Association of Ras and CD68** ^**+**^ **cells in angiogenesis. (A)** Expression of CD34 in breast cancer cells. **(B)** Association of Ras expression with MVD number. **(C)** Association of CD68^+^ cell number with MVD numbers.

Angiogenesis vascularity was defined by MVD assay as previously described [9,10]. CD34 could be detected in all the 120 specimens. All endothelial cells in the tissue were positively stained by CD34 antibodies. The area of greatest vascular density was usually, but not always, at the periphery of the carcinoma. MVD ranged from 4 to 129, with a mean of 46.83. A higher MVD number was correlated with larger tumour size, higher TNM stages, ER positivity and PR positivity. Other histological features had no statistically significant association with MVD number (Table 3). The number of MVD in Ras negative group was 31.77 ± 28.71, compared with 55.25 ± 23.33 in Ras positive group (r = 0.451 *P* = 0.000000299, Figure 4B). Moreover, with regard to the correlation between CD68^+^ cells and MVD, there was a statistically significant correlation between increased number of CD68^+^ cells and increased MVD (r = 0.288 *P* = 0.00146, Figure 4C), indicating the up-regulation of Ras and the infiltration of CD68^+^ macrophages correlated with angiogenesis.

## Disscussion

The Ras family of small guanosine triphosphatases (GTPases), composed of K-, H- and N-Ras, normally transmit signals from cell surface receptors to the interior of the cell. Stimulation of cell surface receptors leads to the activation of guanine exchange factors (GEFs), which, in turn, convert Ras from an inactive GDP-bound state to an active GTP-bound state. In this active state, Ras adopts a conformation that permits effector proteins such as phosphoinositide 3-kinases (PI3Ks), the Rafs mitogen-activated protein kinase kinase kinases, and RalGEFs to bind Ras, leading to their activation and propagation of signaling. This signal is terminated by GTPase-activating proteins (GAPs) that stimulate the GTPase activity of Ras, returning Ras to its inactive GDP-bound state [14].

In most cancers, Ras is inappropriately activated. In fact, 20–30% of all tumors harbor oncogenic point mutations in Ras at Gly^12^, Gly^13^, or Gln^61^ that impair the intrinsic and GAP stimulated GTP hydrolysis, leaving oncogenic Ras in a constitutively active GTP-bound state [22]. If Ras itself is not mutated, often Ras signaling pathway components are inappropriately activated or repressed to promote Ras signaling. Specifically, upstream cell surface receptors that activate Ras, such as epidermal growth factor receptor (EGFR) and the closely related Her-2/Neu, are often amplified or mutated to remain active, resulting in chronic Ras activation [14]. In fewer cases, there is a loss of a GAP, keeping Ras in an active, GTP-bound state [14]. Additionally, downstream activating mutations in Raf and PI3K or loss of the lipid phosphatase PTEN leads to activation of individual Ras effector pathways [14]. Furthermore, expression of Ras was found to be up-regulated in breast cancers [23]. Thus, the Ras pathway could be inappropriately activated in multiple manners.

Tumor-associated macrophage (TAM) represent the major inflammatory component of the stroma of many tumors, able to affect different aspects of the neoplastic tissue [24]. In the tumor microenvironment neoplastic cells shape the differentiation and functional orientation of TAM which, in turn, express several protumoral functions, including secretion of growth factors and matrix-proteases, promotion of angiogenesis and suppression of adaptive immunity [18]. The protumoral role of TAM in cancer is further supported by clinical studies that found a correlation between the high macrophage content of tumors and poor patient prognosis and by evidence showing that long-term use of non-steroidal anti-inflammatory drugs reduces the risk of several cancers [24].

In the present study, we found the association between Ras expression and TAM infiltration by using immunohistochemistry. Mechanically, TAMs are generally recruited from blood monocytes by diverse chemokines such as CCL2 (MCP-1), CCL5, CCL7, CXCL8 and CXCL12, migrate to diverse areas of the tumor microenvironment and differentiate according to surrounding cellular or environmental stimuli [13]. Previous study proved that activation of Ras could result in up-regulation of CCL2 (MCP-1) [15], CCL5 [16,17] and IL-8 [14,18], the widely accepted cytokines participating in the recruitment of monocyte, suggesting the overexpression of Ras could be the stimulation of TAM infiltration. Could overexpression of Ras also be the consequence of TAM infiltration? To verify this hypothesis, we treated breast cancer cells with macrophage-conditional medium (MCM) and evaluate expression of Ras. Interestingly, treatment of MCM repressed Ras expression, rather than overexpressing. Thus, it seems that the overexpression of Ras might not be the consequence of TAM infiltration. The findings in the present study supported the hypothesis that overexpresssion of Ras in breast cancers might recruit infiltration of TAM, which could further promote angiogenesis and cancer progression. Taking together, based on our present investigation and previous studies, we speculated that the infiltration of TAM is probably the consequence but not the inducement of overexpressed Ras.

Angiogenesis (or neovascularization) consists in the formation of new blood vessels from the endothelium of the existing vasculature. It has been demonstrated experimentally that tumor cell proliferation and growth, as well as metastatic spread, is preceded and favored by the formation of new blood vessel [6]. When a new tumor reaches the size of 1–2 mm, its ulterior growth requires the induction of new blood vessels, which may consequently lead to the development of metastases, via the penetration of malignant cells into the circulation [8]. Consequently, angiogenic determination could provide complementary information to that obtained from the tumor biological profile and could thus be used for prognostic assessment and as a therapeutic target in human tumors [25,26]. Quantification of angiogenesis, using microvessel density (MVD) as a parameter, is considered to be a valuable prognostic indicator of breast cancer aggressiveness [27]. Tumour vascularity in invasive breast carcinoma has been extensively investigated in relation to its prognostic significance. Increasing research in angiogenesis has left little doubt about its importance in tumor growth and metastatic dissemination [27].

Both activation of Ras and infiltration of TAM have been found to contribute to the angiogenesis. Expression of oncogenic Ras in tumor cells promotes survival of endothelial cells, while loss of Ras expression triggered apoptosis in endothelial cells, collapse in tumor vasculature, and regression of the tumor [28]. Moreover, Ras promotes transcription of vascular endothelial growth factor (VEGF), a key factor promoting angiogenesis [29]. Ras also upregulates the expression of the cytokines IL-8 and IL-6, both of which promote angiogenesis [22].

Macrophages can exert a dual influence on blood vessel formation and function. In general, as for interaction with neoplastic cells, the pro-angiogenic functions of TAM prevail. In several studies, TAM accumulation has been associated with angiogenesis and with the production of angiogenic factors such as VEGF and platelet-derived endothelial cell growth factor. TAM tends to accumulate in hypoxic regions of tumors. A number of pro-angiogenesis molecules have been shown to be expressed by macrophage in low oxygen conditions, such as VEGF, TNF-α and IL-8. Therefore, macrophages recruited in situ represent an indirect pathway of amplification of angiogenesis, in concert with angiogenic molecules directly produced by tumor cells [24].

In conclusion, inflammatory microenvironment has been proved to promote cancerous angiogenesis. Previous studies have proved the relationship between oncogenic Ras and angiogenesis, as well as the association between infiltration of TAM and neovascularization. Our present study found the link between oncogenic Ras, infiltration of TAM and formation of neovascularization. Thus, our investigation provided a new hypothesis to reveal the mechanism involved in the oncogenic Ras modified inflammatory microenvironment which further promotes angiogenesis and cancer progression.

## Abbreviations

TAM: Tumor-associated macrophages
MVD: Microvessel density
MCSF: Macrophage colony-stimulating-factor
MCM: Macrophage-conditioned medium
IL-8: Interleukin 8
UICC: International Union Against Cancer
TNM: Tumor/lymph node/metastasis
OS: Overall survival
VEGF: Vascular endothelial growth factor
PBS: Phosphate-buffered saline
BSA: Bull serum albumin
FCS: Fetal calf serum
ER: Estrogen receptor
PR: Progesterone receptor
GTPase: Guanosine triphosphatase
GEF: Guanine exchange factor
PI3K: Phosphoinositide 3-kinase
GAP: GTPase-activating protein
EGFR: Epidermal growth factor receptor
